# Open-skill vs. closed-skill sports and executive function in children and adolescents: a mini review of mechanisms and moderating factors

**DOI:** 10.3389/fpsyg.2026.1873432

**Published:** 2026-06-30

**Authors:** Huiyan Zhang, Wei Wei, Weiwei Zhang

**Affiliations:** 1 Hainan Tropical Ocean University, Sanya, China; 2 New Era University, Selangor, Malaysia; 3 Zhengzhou College of Finance and Economics, Zhengzhou, China; 4 Beijing College of Finance and Commerce, Beijing, China

**Keywords:** adolescents, closed-skill exercise, cognitive flexibility, executive function, inhibitory control, neuroplasticity, open-skill exercise, working memory

## Abstract

Executive function (EF), comprising inhibitory control, working memory, and cognitive flexibility, undergoes critical maturation during childhood and adolescence and predicts academic achievement, social adjustment, and mental health. Although physical exercise is now broadly accepted as a cognitive enhancer, growing evidence suggests that the type of motor activity matters: open-skill sports (e.g., basketball, badminton), performed in unpredictable, externally-paced environments demanding rapid perception–action coupling, may differ from closed-skill sports (e.g., running, swimming), performed at self-selected pace in stable settings, in their cognitive yields. This mini-review synthesizes recent evidence on (a) the differential effects of open- vs. closed-skill exercise on EF subcomponents in children and adolescents, (b) the underlying neurophysiological and molecular mechanisms, (c) moderators including dose parameters and individual characteristics, and (d) ongoing controversies and methodological gaps. Open-skill sports tend to confer larger benefits for inhibitory control and cognitive flexibility through enhanced conflict monitoring and improved neural efficiency, whereas closed-skill sports often outperform them on working memory and on hyperactivity/impulsivity in attention-deficit/hyperactivity disorder (ADHD). Findings are, however, not uniform, and some syntheses report no significant overall difference between sport types, indicating that any advantage is domain-specific rather than absolute. These benefits are dose-dependent and moderated by age, sex, and baseline cognition. Future work should employ longitudinal designs, finer-grained sport taxonomies that move beyond the open–closed dichotomy, individualized intervention prescriptions, and multimodal neuroimaging to clarify the neural and molecular pathways linking exercise type to EF development.

## 1 Introduction

Executive function (EF) refers to a constellation of higher-order cognitive processes, which are most prominently inhibitory control, working memory, and cognitive flexibility, that support goal-directed behavior, academic achievement, social (Best and Miller, 2010).

adjustment, and mental health (Diamond, 2013). Childhood and adolescence are periods of pronounced EF maturation, during which the prefrontal cortex and its associated networks are highly responsive to environmental input. Identifying modifiable lifestyle factors that scaffold EF development is therefore a priority for cognitive neuroscience and educational practice. A clinically important case is attention-deficit/hyperactivity disorder (ADHD): relative to typically developing peers, children and adolescents with ADHD show marked deficits in inhibitory control and working memory linked to altered fronto-striatal function, which makes this group both a priority target for intervention and an informative model for probing exercise–EF relations (Huang et al., 2023).

Among such factors, regular physical exercise has attracted substantial attention. Crucially, the cognitive yield of exercise depends not only on intensity and duration, but also on the *type* of motor skill involved. Sports are commonly classified along an open–closed continuum based on environmental predictability: open-skill sports (e.g., basketball, football, and badminton) require continuous adjustment to a dynamic, externally-paced environment, whereas closed-skill sports (e.g., running, swimming, and athletics) are performed in stable, self-paced settings with predefined movement patterns (Contreras-Osorio et al., 2022; Zhu et al., 2023). This taxonomy provides a useful framework for examining how different motor demands map onto cognitive outcomes.

A consistent literature now indicates that physical activity benefits children's EF, but the magnitude and even the direction of these effects vary by sport type. Some studies report that open-skill sports yield greater EF gains because they impose continuous perceptual-cognitive-motor demands (Shih et al., 2025; Zhu et al., 2023), whereas others find selective advantages of closed-skill exercise on specific subcomponents such as working memory and the regulation of ADHD symptoms (Feng et al., 2023; Huang et al., 2023; Qiu et al., 2024). These divergent findings have prompted active debate and underscore the need to clarify the cognitive, neural, and dose–response mechanisms involved. This mini-review summarizes recent evidence on the differential effects of open- and closed-skill exercise on adolescent EF, integrates underlying neurophysiological mechanisms, identifies moderators, and outlines unresolved controversies and future directions.

## 2 Search strategy

We searched PubMed, Web of Science, Scopus, and PsycINFO for English-language articles published between January 2020 and March 2026, using combinations of the following terms in title, abstract, and keyword fields: (“open-skill” OR “closed-skill” OR “interceptive” OR “strategic” OR “sequential” OR “self-paced” OR “externally-paced”) AND (“exercise” OR “sport^*^” OR “physical activity” OR “training”) AND (“executive function^*^” OR “inhibitory control” OR “working memory” OR “cognitive flexibility” OR “attention”) AND (“child^*^” OR “adolescent^*^” OR “youth” OR “schoolchildren” OR “students”).

Because the open–closed taxonomy has only recently become a focus of developmental cognitive neuroscience, priority was given to original studies, randomized controlled trials, systematic reviews, and meta-analyses published within the last 5 years; foundational older work was retained when conceptually necessary. Reference lists of retrieved reviews were hand-searched for additional eligible studies. The primary inclusion population comprised children and adolescents (5–18 years) participating in identifiable open- or closed-skill exercise and assessed on at least one EF outcome. Studies focused exclusively on motor performance without EF outcomes, conference abstracts, and non-peer-reviewed gray literature were excluded. Where developmental data were sparse, a limited number of young-adult (e.g., university) and clinical (ADHD) studies were retained solely for their mechanistic or methodological value; such studies are explicitly identified as such in the text and were not pooled with the developmental (5–18 years) evidence. Records were screened by the first author (H.Z.) against these predefined criteria. Because a mini-review is a narrative rather than a systematic synthesis, full duplicate (proportional) screening by a second reviewer is not mandated by the article type; nevertheless, to reduce the risk of selective inclusion, a co-author independently re-checked the eligibility decisions, and any disagreements were resolved by discussion. Consistent with the narrative scope of a mini-review, no formal risk-of-bias instrument was applied; instead, the methodological quality of the primary studies (e.g., study design, sample size, and the validity of the EF measures employed) was appraised qualitatively and informed the weight given to individual findings throughout Sections 3–6. Single-primary-screener selection and the absence of a standardized critical-appraisal tool are acknowledged as limitations (Section 6.3).

## 3 Differential effects of open- and closed-skill sports on adolescent EF

### 3.1 Overall effects on executive function

Network meta-analyses indicate that diverse exercise modalities improve EF in children and adolescents, but open-skill sports often emerge with the largest pooled effects. Zhu et al. (2023) reviewed 59 studies in children and adolescents with ADHD and reported a substantially larger overall effect for open-skill exercise (SMD = 1.96) than for closed-skill exercise, with the most pronounced advantage for inhibitory control. In typically developing children, Feng et al. (2023) likewise documented superior gains from open- and sequential-skill activities, with open-skill exercise producing the largest improvements on working memory (SMD = −0.833) and sequential-skill exercise yielding stronger effects on inhibitory control and cognitive flexibility (Table 1).

**Table 1:** Representative evidence comparing open- and closed-skill sports for executive function in children and adolescents.

Study	Design and population	Sport/exercise comparison	Executive-function domain(s)	Principal findings	Interpretive value for the mini-review
Zhu et al. (2023)	Systematic review and network meta-analysis; children and adolescents with ADHD	Multiple exercise modalities, including open- and closed-skill exercise	Overall EF; inhibitory control; working memory; cognitive flexibility	Open-skill exercise showed a larger overall pooled effect than closed-skill exercise, with the clearest advantage for inhibitory control; closed-skill aerobic exercise ranked highly for working memory.	Supports a domain-specific model: open-skill activities may preferentially enhance inhibition, whereas closed-skill aerobic training may be useful for working-memory outcomes.
Feng et al. (2023)	Meta-analysis; typically developing children	Open-, closed-, sequential-, and continuous-skill exercise interventions	Inhibitory control; working memory; cognitive flexibility	Open-skill exercise produced marked working-memory gains, while sequential-skill activities were especially effective for inhibition and flexibility. Long-term, moderate-intensity protocols were more favorable than acute high-intensity bouts.	Shows that the open-closed distinction is useful but incomplete; action structure and dose parameters also shape EF gains.
Huang et al. (2023)	Meta-analysis; children and adolescents with ADHD	Chronic exercise modalities, including open- and closed-skill exercise	Core ADHD symptoms; EF-related symptoms	Closed-skill exercise more effectively reduced core ADHD symptoms, particularly hyperactivity/impulsivity, than open-skill exercise.	Balances the open-skill advantage by showing that closed-skill exercise may be clinically preferable for symptom regulation in ADHD.
Shih et al. (2025)	Large observational analysis using the ABCD Study; 9–10-year-old children	Open-skill sport participants, closed-skill sport participants, and non-athletes	Inhibitory control; working memory	Children participating in open-skill sports outperformed closed-skill peers and non-athletes on Flanker and List-Sorting Working Memory tasks.	Provides large-sample developmental evidence that open-skill sports are associated with superior EF performance in late childhood.
Contreras-Osorio et al. (2022)	School-based intervention; schoolchildren	Handball as open-skill training vs. athletics as closed-skill training	Inhibitory control; semantic fluency; broader EF	Both sport types improved EF, but handball preferentially improved inhibitory control, whereas athletics enhanced semantic fluency.	Suggests that open- and closed-skill sports can both be beneficial, with effects depending on the EF subcomponent assessed.
De Waelle et al. (2021)	Cross-sectional study; children aged 8–12 years	Team sports vs. self-paced sports and non-athlete comparison	Composite EF; especially cognitive flexibility	Children involved in team sports showed superior EF, with the most pronounced advantage for cognitive flexibility.	Highlights the role of interpersonal, strategic, and rapidly changing environments in supporting flexible cognition.
Takahashi and Grove (2023)	Acute experimental study with fNIRS	10-min badminton bout as open-skill exercise vs. running as closed-skill exercise	Inhibitory control; prefrontal activation	Acute badminton improved Stroop performance without a parallel increase in prefrontal activation; running did not produce the same behavioral benefit.	Supports a neural-efficiency explanation for acute open-skill benefits on inhibition.
Ludyga et al. (2022a)	Neurophysiological study; children and adolescents	Long-term open-skill vs. closed-skill sport participation	Interference control; response inhibition; ERP indices	Open-skill participants showed larger N200 and N450 amplitudes during conflict tasks, indicating more efficient conflict monitoring and resolution.	Provides mechanistic ERP evidence that open-skill exposure affects early conflict-detection processes.
Gu et al. (2024)	Randomized controlled trial; female adolescents with subthreshold depression	Eight-week open-skill vs. closed-skill exercise intervention	Cognitive flexibility; working memory; depressive symptoms	Both exercise types reduced subthreshold depression; closed-skill exercise yielded more durable gains in cognitive flexibility and working memory.	Shows that psychological status and follow-up timing can modify which sport type appears superior.
Qiu et al. (2024)	Systematic review and meta-analysis; individuals with ADHD	Closed- vs. open-skill exercise	Overall EF; inhibition; cognitive flexibility; working memory	Closed-skill exercise outperformed open-skill exercise for overall EF in ADHD, although open-skill exercise produced the largest single inhibitory-control effect.	Indicates that clinical populations may require individualized prescriptions rather than a simple open-skill preference.
Heilmann et al. (2022)	Systematic review and meta-analysis; athletes across sport types	Open- vs. closed-skill sport practice	Executive functions across domains	No significant overall difference was found between open- and closed-skill athletes.	Provides a key counterpoint: binary sport classification may be too coarse to capture cognitive-demand differences.
Li et al. (2024)	16-week quasi-experimental study; university students	Football as open-skill sport vs. golf as closed-skill sport	Inhibitory control	Football and golf produced equivalent gains in inhibitory control despite different open-closed classifications.	Demonstrates that some closed-skill sports impose substantial strategic and attentional demands, challenging the simple taxonomy.
Sun et al. (2025)	Prospective longitudinal cohort protocol; elite youth athletes	Open-skill water polo vs. closed-skill swimming	Developmental trajectories of EF	The planned 5-year design aims to disentangle training effects from maturation and selection effects.	Points to the type of longitudinal evidence needed to resolve causal uncertainty in the field.

However, this pattern is not universal. Huang et al. (2023) reported that closed-skill exercise more effectively reduced core ADHD symptoms, particularly hyperactivity/impulsivity (SMD = −0.83), than did open-skill exercise. These inconsistencies likely reflect heterogeneity in sample characteristics, intervention parameters, and outcome measures, and they suggest that the open–closed advantage is domain-specific rather than absolute.

### 3.2 Subcomponent specificity

#### 3.2.1 Inhibitory control

Open-skill sports show consistent superiority for inhibitory control. In a large analysis of 9–10-year-olds from the Adolescent Brain Cognitive Development (ABCD) Study, open-skill participants outperformed closed-skill participants and non-athletes on the Flanker task and the List-Sorting Working Memory task (Shih et al., 2025). Neurophysiological work corroborates this advantage: open-skill athletes display larger N200 and N450 amplitudes during conflict tasks, indicating more efficient neural processing of interference (Ludyga et al., 2022a).

#### 3.2.2 Working memory

Closed-skill exercise may carry distinct value for working memory. Zhu et al. (2023) reported that aerobic-dominant closed-skill exercise had the highest probability of being optimal for working memory (SUCRA = 75.9%). Judo training improved working memory capacity in children with ADHD and enhanced contralateral delay activity, suggesting more efficient maintenance processes (Ludyga et al., 2022b). Yet other work finds open-skill athletes outperforming closed-skill peers on working memory tasks (Ren et al., 2025), again pointing to task- and population-specific effects.

#### 3.2.3 Cognitive flexibility

Multimodal exercise integrating both open- and closed-skill components appears most effective for cognitive flexibility (Zhu et al., 2023). De Waelle et al. (2021) found that 8–12-year-olds in team sports outperformed self-paced athletes and non-athletes on a composite EF battery, with the most pronounced benefits for cognitive flexibility, plausibly reflecting the constant decision-making and strategy-switching demands of team play.

### 3.3 Acute vs. chronic interventions

Acute and chronic exercise act through partially distinct mechanisms. A single 10-min bout of badminton (open-skill) improved Stroop performance without increasing prefrontal cortex activation, suggesting enhanced *neural efficiency*, whereas an equivalent running bout (closed-skill) produced no such acute benefit (Takahashi and Grove, 2023). Long-term interventions show more nuanced patterns. A 12-week study comparing handball and athletics in schoolchildren found that both improved EF, but with subcomponent-specific adaptations: athletics enhanced semantic fluency, whereas handball improved inhibitory control (Contreras-Osorio et al., 2022). Eight weeks of either open- or closed-skill exercise reduced subthreshold depression in female adolescents, with closed-skill exercise yielding more durable cognitive flexibility and working memory gains (Gu et al., 2024). Long-term effects, therefore, appear to depend on the match between sport type and the targeted EF subcomponent.

## 4 Neurophysiological and molecular mechanisms

### 4.1 Event-related potential evidence

ERP studies illuminate when, in the cognitive processing stream, sport type confers an advantage. Long-term open-skill participants exhibit larger Stroop P200 amplitudes, indicating enhanced early attentional resource allocation (Zhou et al., 2020). For conflict-related components, open-skill exposure correlates with larger N200 and N450 amplitudes in 9–14-year-olds (Ludyga et al., 2022a), reflecting more efficient conflict monitoring and resolution. P300 amplitude, typically associated with stimulus evaluation, does not consistently differ by sport type, suggesting the open-skill advantage operates primarily at early conflict-detection rather than later allocation stages. Within open-skill sports, interceptive athletes (e.g., basketball) show shorter reaction times in interference conditions, while strategic athletes (e.g., football) demonstrate greater early-stage resilience capacity (Wang C. H. et al., 2023), implying meaningful within-category heterogeneity.

### 4.2 Functional neuroimaging

Functional near-infrared spectroscopy (fNIRS) data converge with the neural-efficiency hypothesis: although acute badminton improved Stroop performance, prefrontal activation did not differ from acute running, implying that better behavioral performance occurred at comparable or lower neural cost (Takahashi and Grove, 2023). At the structural level, ABCD analyses indicate that organized sport participation, irrespective of type, is associated with greater subcortical gray matter volume and broad white-matter integrity, alongside cognitive benefits, with no robust team-vs.-individual contrast (Tan et al., 2025). This pattern implies broadly non-specific structural effects of organized sport that may be distinct from the EF-specific effects of cognitive load.

### 4.3 Exercise-induced neuroplasticity

At the molecular level, exercise acts through exerkines, bioactive molecules released during physical activity, including brain-derived neurotrophic factor (BDNF) and cathepsin B, that modulate synaptic plasticity and adult neurogenesis (Vints et al., 2022). Comparing young-adult fencers (open-skill) and swimmers (closed-skill), Gökçe et al. (2021) reported higher cathepsin B levels and stronger acute-exercise responsiveness in open-skill athletes, while baseline BDNF did not differ between groups. The cognitive benefits of open-skill sports may therefore derive partly from cognitively demanding load-induced plasticity rather than aerobic adaptation alone.

## 5 Moderating factors

### 5.1 Exercise dose

In typically developing children, long-term, moderate-intensity protocols outperform acute, high-intensity protocols, with long-term interventions producing significant working-memory gains and moderate intensity benefiting all three EF subcomponents (Feng et al., 2023). For ADHD, longitudinal open-skill exercise of ≥60 min per session, twice weekly, optimally improves inhibitory control (Wang M. et al., 2023). Open-skill interventions of 6–10 weeks appear most effective for working memory, while sessions of 75–120 min maximize cognitive flexibility gains (Hu et al., 2024). Cognitive benefits emerge at session durations beyond approximately 30 min but may plateau (Song et al., 2022), suggesting a non-linear dose–response relationship.

### 5.2 Individual differences

Age. Open-skill exercise benefits inhibitory control most strongly in 5–9-year-olds and working memory most strongly in 10–16-year-olds (Hu et al., 2024), aligning with the developmental trajectories of these subcomponents (Albaladejo-García et al., 2023).

Sex. A large meta-regression found exercise–cognition benefits to be smaller in females than in males, with sex-dependent dose–response curves (Ludyga et al., 2020), although other studies report no sex moderation (Spanou et al., 2022).

Baseline cognition. Children with EF deficits, such as those with ADHD, may derive particularly large gains. In ADHD, Qiu et al. (2024) found closed-skill exercise to outperform open-skill exercise on overall EF (inhibitory control SMD = −1.00; cognitive flexibility SMD = −1.33; working memory SMD = −0.85), although open-skill exercise produced the single largest inhibitory-control effect (SMD = −1.98).

### 5.3 Sport sub-typology

The open–closed dichotomy can be refined. Within open-skill sports, strategic sports (e.g., basketball, football) demand greater decision-making and tactical flexibility than interceptive sports (e.g., badminton, table tennis), and produce larger inhibitory-control gains (SMD = −0.707; Hu et al., 2024). Within action structure, sequential skills (e.g., martial-arts forms, gymnastics) outperform continuous skills on inhibition and flexibility (Feng et al., 2023), and open-sequential combinations yield the largest EF benefits overall (Shi et al., 2022).

## 6 Controversies and limitations

### 6.1 Open-skill advantage vs. null findings

The “open-skill advantage” account (Ren et al., 2025; Shih et al., 2025; Zhu et al., 2023) is balanced by null findings. Heilmann et al. (2022) pooled 19 studies and reported no significant overall difference between open- and closed-skill athletes (Hedges' *g* = 0.174, *p* = 0.157). In a sample of 230 elite athletes, sport type produced a significant main effect on only one EF measure, with most cognitive scores within population norms (Vona et al., 2024). These results imply that the binary open–closed distinction may oversimplify the cognitive-demand landscape.

### 6.2 Classification heterogeneity and methodological limits

The dichotomous taxonomy is based on environmental predictability, but sport-related cognitive demand also encompasses decision complexity, time pressure, perceptual load, and social interaction. Golf, formally a closed-skill sport, imposes considerable strategic and attentional demand: 16 weeks of either football or golf training in university students yielded equivalent gains in inhibitory control (Li et al., 2024). Variability in participants, intervention parameters, EF instruments, and study designs (with cross-sectional designs predominating) further limits direct comparability (Sun et al., 2025).

### 6.3 Limitations of the current evidence base

Cross-sectional designs cannot disambiguate selection from training effects (Kalén et al., 2021). Sample sizes are typically small, follow-up is short, and most studies rely on laboratory-based EF tasks of limited ecological validity. Recent work using ecological EF measures has linked open-skill exercise to reduced adolescent smartphone dependence *via* improvements in ecological inhibition and working memory (Xie et al., 2025; Zhang et al., 2026), but such ecologically grounded studies remain rare. Neuroimaging work, in turn, has focused largely on the prefrontal cortex, with comparatively little attention to whole-brain network connectivity or subcortical contributions.

Limitations of the present review should also be acknowledged. As a narrative mini-review, it did not apply a formal risk-of-bias tool or a standardized critical-appraisal instrument, and no quantitative synthesis or sensitivity analysis was performed; its conclusions therefore rest on a qualitative weighing of evidence that is more vulnerable to selection and interpretation bias than a full systematic review. Inclusion was restricted to English-language reports and relied on a single primary screener, raising the possibility of language, publication, and selection bias. Critically, the evidence base itself is dominated by cross-sectional and short-term studies that use heterogeneous sport classifications and EF instruments, so the reported open-vs. closed-skill contrasts may partly reflect differences in study design and measurement rather than genuine cognitive differences. The open–closed dichotomy may also conflate several distinct cognitive demands (decision complexity, time pressure, perceptual load, and social interaction), and the comparative effect sizes drawn from different meta-analyses are not directly commensurable. These constraints temper the strength of any causal or prescriptive claims and should be weighed when interpreting the syntheses summarized above.

## 7 Future directions

Longitudinal and causally-informative designs. A planned 5-year prospective cohort comparing elite open-skill (water polo) and closed-skill (swimming) youth athletes will be valuable for disentangling training and maturation effects (Sun et al., 2025). Cross-lagged panel models can further test bidirectional EF–exercise relations and characterize reciprocal feedback between cognitive growth and sport participation.

Refined taxonomies and individualized prescriptions. Multidimensional cognitive-demand frameworks are needed beyond the open-closed binary (Heilmann et al., 2022; Li et al., 2024). For 5–9-year-olds, prioritizing open-skill exercise to support inhibitory development is reasonable; for adolescents, integrating closed-skill aerobic training to support working memory may be advisable (Hu et al., 2024). For children with ADHD, multimodal protocols of ≥12 weeks, ≥2 sessions per week at moderate-to-vigorous intensity that combine open- and closed-skill elements, are indicated (Qiu et al., 2024).

Multimodal neuroimaging and computational modeling. Combining EEG, fMRI, and fNIRS, supplemented by diffusion tensor imaging for white-matter microstructure and resting-state functional connectivity for network reorganization, will yield a more complete neural account (Ludyga et al., 2022a; Takahashi and Grove, 2023; Tan et al., 2025). Systems factorial technology and computational cognitive modeling can resolve which processing stages benefit most from open-skill training (Wang C. H. et al., 2023). Linking exerkine dynamics to neural and behavioral outcomes will help build a cross-level mechanistic model of exercise-induced cognitive plasticity (Gökçe et al., 2021; Vints et al., 2022).

## 8 Conclusion

Open- and closed-skill sports both promote adolescent EF, but with distinct profiles: open-skill exercise tends to favor inhibitory control and cognitive flexibility through enhanced conflict monitoring and improved neural efficiency, whereas closed-skill exercise often yields stronger working memory gains and reductions in hyperactivity/impulsivity. These effects are shaped by exercise dose, age, sex, baseline cognition, and the strategic, interceptive, continuous, or sequential character of the activity. Resolving outstanding controversies will require longitudinal designs, finer-grained taxonomies, individualized prescriptions, and multimodal neuroimaging. Such advances will provide a stronger evidence base to inform educational, sports, and public-health practice for adolescent cognitive health. For physical-education teachers and youth coaches, the current evidence supports a varied, age-tailored programme rather than a single best sport: emphasizing open-skill, decision-rich games (e.g., basketball, badminton) to challenge inhibitory control and cognitive flexibility, while retaining closed-skill aerobic activity (e.g., running, swimming) to support working memory and, in children with ADHD, to help regulate hyperactivity and impulsivity. Sessions of at least 30 min at moderate intensity, performed two or more times per week and sustained across several weeks with gradual increases in cognitive and tactical demand, provide a practical starting point that can be individualized to each child's age and baseline ability. Given the limitations noted above, these suggestions should be regarded as provisional guidance rather than prescriptive rules.

## References

[B1] Albaladejo-García C. García-Aguilar F. Moreno F. J. (2023). The role of inhibitory control in sport performance: systematic review and meta-analysis in stop-signal paradigm. Neurosci. Biobehav. Rev. 147:105108. doi: 10.1016/j.neubiorev.2023.105108 36828162

[B2] Best J. R. Miller P. H. (2010). A developmental perspective on executive function. Child Dev. 81, 1641–1660. doi: 10.1111/j.1467-8624.2010.01499.x 21077853 PMC3058827

[B3] Contreras-Osorio F. Guzmán-Guzmán I. P. Cerda-Vega E. Chirosa-Ríos L. Ramírez-Campillo R. Campos-Jara C. (2022). Effects of the type of sports practice on the executive functions of schoolchildren. Int. J. Environ. Res. Public Health 19:3886. doi: 10.3390/ijerph19073886 35409571 PMC8998109

[B4] De Waelle S. Laureys F. Lenoir M. Bennett S. J. Deconinck F. J. A. (2021). Children involved in team sports show superior executive function compared to their peers involved in self-paced sports. Children (Basel) 8:264. doi: 10.3390/children8040264 33808250 PMC8065925

[B5] Diamond A. (2013). Executive functions. Annu. Rev. Psychol. 64, 135–168. doi: 10.1146/annurev-psych-113011-143750 23020641 PMC4084861

[B6] Feng X. Zhang Z. Jin T. Shi P. (2023). Effects of open and closed skill exercise interventions on executive function in typical children: a meta-analysis. BMC Psychol. 11:420. doi: 10.1186/s40359-023-01317-w 38037184 PMC10690989

[B7] Gökçe E. Güneş E. Ari F. Hayme S. Nalçaci E. (2021). Comparison of the effects of open- and closed-skill exercise on cognition and peripheral proteins: a cross-sectional study. PLoS ONE 16:e0251907. doi: 10.1371/journal.pone.0251907 34086693 PMC8177547

[B8] Gu Q. Zhao X. Lin L. Teo W. P. Liu L. Yuan S. (2024). Effects of open-skill and closed-skill exercise on subthreshold depression in female adolescents: a randomized controlled trial. Int. J. Clin. Health Psychol. 24:100512. doi: 10.1016/j.ijchp.2024.100512 39659958 PMC11630631

[B9] Heilmann F. Weinberg H. Wollny R. (2022). The impact of practicing open- vs. closed-skill sports on executive functions—a meta-analytic and systematic review with a focus on characteristics of sports. Brain Sci. 12:1071. doi: 10.3390/brainsci12081071 36009134 PMC9406193

[B10] Hu S. Shi P. Zhang Z. Feng X. Zhang K. Jin T. (2024). Effects of open-skill exercise on executive functions in children and adolescents: a systematic review and meta-analysis. Front. Hum. Neurosci. 18:1495371. doi: 10.3389/fnhum.2024.1495371 39967690 PMC11832504

[B11] Huang H. Jin Z. He C. Guo S. Zhang Y. Quan M. (2023). Chronic exercise for core symptoms and executive functions in ADHD: a meta-analysis. Pediatrics 151:e2022057745. doi: 10.1542/peds.2022-057745 36510746

[B12] Kalén A. Bisagno E. Musculus L. Raab M. Pérez-Ferreirós A. Williams A. M. . (2021). The role of domain-specific and domain-general cognitive functions and skills in sports performance: a meta-analysis. Psychol. Bull. 147, 1290–1308. doi: 10.1037/bul0000355 35404636

[B13] Li Y. F. Gao T. Luo L. P. He S. (2024). Comparative effects of open-skill and closed-skill sports on executive function in university students: a 16-week quasi-experimental study. Front. Psychol. 15:1457449. doi: 10.3389/fpsyg.2024.1457449 39434911 PMC11491408

[B14] Ludyga S. Gerber M. Pühse U. Looser V. N. Kamijo K. (2020). Systematic review and meta-analysis investigating moderators of long-term effects of exercise on cognition in healthy individuals. Nat. Hum. Behav. 4, 603–612. doi: 10.1038/s41562-020-0851-8 32231280

[B15] Ludyga S. Mücke M. Andrä C. Gerber M. Pühse U. (2022a). Neurophysiological correlates of interference control and response inhibition processes in children and adolescents engaging in open- and closed-skill sports. J. Sport Health Sci. 11, 224–233. doi: 10.1016/j.jshs.2021.01.001 33421617 PMC9068557

[B16] Ludyga S. Mücke M. Leuenberger R. Bruggisser F. Pühse U. Gerber M. . (2022b). Behavioral and neurocognitive effects of judo training on working memory capacity in children with ADHD: a randomized controlled trial. NeuroImage Clin. 36:103156. doi: 10.1016/j.nicl.2022.103156 35988343 PMC9402389

[B17] Qiu C. Zhai Q. Chen S. (2024). Effects of practicing closed- vs. open-skill exercises on executive functions in individuals with attention deficit hyperactivity disorder (ADHD)—a meta-analysis and systematic review. Behav. Sci. (Basel) 14:499. doi: 10.3390/bs14060499 38920831 PMC11200859

[B18] Ren S. Shi P. Feng X. Zhang K. Wang W. (2025). Executive function strengths in athletes: a systematic review and meta-analysis. Brain Behav. 15:e70212. doi: 10.1002/brb3.70212 39740775 PMC11688047

[B19] Shi P. Tang Y. Zhang Z. Feng X. Li C. (2022). Effect of physical exercise in real-world settings on executive function of typical children and adolescents: a systematic review. Brain Sci. 12:1734. doi: 10.3390/brainsci12121734 36552193 PMC9775424

[B20] Shih C. H. Broadnax M. Eckner J. Veliz P. Varangis E. (2025). Cognitive benefits of open-skill sports in childhood: evidence from the ABCD study. Med. Sci. Sports Exerc. 57, 1182–1188. doi: 10.1249/MSS.0000000000003655 39876084

[B21] Song W. Feng L. Wang J. Ma F. Chen J. Qu S. . (2022). Play smart, be smart? Effect of cognitively engaging physical activity interventions on executive function among children 4–12 years old: a systematic review and meta-analysis. Brain Sci. 12:762. doi: 10.3390/brainsci12060762 35741648 PMC9220861

[B22] Spanou M. Stavrou N. Dania A. Venetsanou F. (2022). Children's involvement in different sport types differentiates their motor competence but not their executive functions. Int. J. Environ. Res. Public Health 19:5646. doi: 10.3390/ijerph19095646 35565039 PMC9103227

[B23] Sun P. Tang Q. Yang J. Li N. Chen J. Cheng W. . (2025). Effects of open-skill and closed-skill sports on the development of executive function: protocol for a prospective longitudinal cohort study in elite youth athletes. BMJ Open Sport Exerc. Med. 11:e002730. doi: 10.1136/bmjsem-2025-002730 40546752 PMC12182203

[B24] Takahashi S. Grove P. M. (2023). Impact of acute open-skill exercise on inhibitory control and brain activation: a functional near-infrared spectroscopy study. PLoS ONE 18:e0276148. doi: 10.1371/journal.pone.0276148 36961779 PMC10038255

[B25] Tan F. M. Yu J. Goodwill A. M. (2025). Sports participation and childhood neurocognitive development. Dev. Cogn. Neurosci. 71:101492. doi: 10.1016/j.dcn.2024.101492 39740341 PMC11750462

[B26] Vints W. A. J. Levin O. Fujiyama H. Verbunt J. Masiulis N. (2022). Exerkines and long-term synaptic potentiation: mechanisms of exercise-induced neuroplasticity. Front. Neuroendocrinol. 66:100993. doi: 10.1016/j.yfrne.2022.100993 35283168

[B27] Vona M. de Guise É. Leclerc S. Deslauriers J. Romeas T. (2024). Multiple domain-general assessments of cognitive functions in elite athletes: contrasting evidence for the influence of expertise, sport type and sex. Psychol. Sport Exerc. 75:102715. doi: 10.1016/j.psychsport.2024.102715 39048061

[B28] Wang C. H. Fu H. L. Kao S. C. Moreau D. Yang C. T. (2023). Systems factorial technology provides novel insights into the cognitive processing characteristics of open-skill athletes. Psychol. Sport Exerc. 66:102395. doi: 10.1016/j.psychsport.2023.102395 37665857

[B29] Wang M. Yang X. Yu J. Zhu J. Kim H. D. Cruz A. (2023). Effects of physical activity on inhibitory function in children with attention deficit hyperactivity disorder: a systematic review and meta-analysis. Int. J. Environ. Res. Public Health 20:1032. doi: 10.3390/ijerph20021032 36673793 PMC9859519

[B30] Xie C. Zhang Z. Wang S. Zhang J. Jin Y. (2025). Effects of community-based open and closed skill exercise interventions on ecological executive function and mobile phone dependence of adolescents. Front. Psychol. 16:1694637. doi: 10.3389/fpsyg.2025.1694637 41459289 PMC12738362

[B31] Zhang Y. Hua R. Deng M. Shi P. (2026). The correlation between exercise types and adolescents' executive function and mobile phone dependence: a cross-sectional study from the perspective of motor skill classification. Front. Psychol. 17:1695406. doi: 10.3389/fpsyg.2026.1695406 41725667 PMC12920566

[B32] Zhou F. Xi X. Qin C. (2020). Regular open-skill exercise generally enhances attentional resources related to perceptual processing in young males. Front. Psychol. 11:941. doi: 10.3389/fpsyg.2020.00941 32508721 PMC7248399

[B33] Zhu F. Zhu X. Bi X. Kuang D. Liu B. Zhou J. . (2023). Comparative effectiveness of various physical exercise interventions on executive functions and related symptoms in children and adolescents with attention deficit hyperactivity disorder: a systematic review and network meta-analysis. Front. Public Health 11:1133727. doi: 10.3389/fpubh.2023.1133727 37033046 PMC10080114

